# Open plate fixation in displaced pediatric proximal humerus fractures is safe and leads to very good functional outcomes

**DOI:** 10.1007/s00402-024-05526-9

**Published:** 2024-09-15

**Authors:** Sebastian Oenning, Philipp A. Michel, Imke Vehring, Lukas Heilmann, J. Christoph Katthagen, Michael J. Raschke

**Affiliations:** 1 Department of Trauma, Hand and Reconstructive Surgery, University Hospital Muenster, Waldeyer Straße 1, 48149 Muenster, Germany; 2 Department of Trauma Surgery and Orthopedics, University Medical Center Hamburg-Eppendorf, Neues Klinikum O10, Martinistraße 52, 20246 Hamburg, Germany

**Keywords:** Proximal humerus fracture, Pediatric trauma, Children, Open reduction, Internal fixation, Plate fixation, Soft tissue interposition

## Abstract

**Introduction:**

In displaced pediatric proximal humerus fractures (PHF), surgical treatment ranges from closed to open procedures. Soft tissue interposition can impede closed reduction, making open techniques necessary. While K-wire fixation and elastic stable intramedullary nailing (ESIN) lead to good results, plate fixation could be an alternative in patients with limited growth potential and highly unstable or insufficiently retained fractures. Only few studies with low sample sizes have assessed plate fixation, yet. In this study, the outcome of pediatric PHFs treated with plate fixation was evaluated.

**Materials and methods:**

We present a retrospective case series of 18 patients with open growth plates and unilateral, displaced PHFs, treated with plate fixation. The mean age at trauma was 12.1 years (± 2.4), the mean follow-up was 6.52 years (± 4.37). A mean fracture angulation of 32.3° (± 10.89°) was seen. Postoperative assessments included range of motion, clinical scores [Simple Shoulder Test (SST), Subjective Shoulder Value (SSV), American Shoulder and Elbow Surgeons (ASES) Shoulder Score, Pediatric/Adolescent Shoulder Score (PASS), Disabilities of Arm, Shoulder and Hand (DASH) Score], radiological parameters and subjective satisfaction.

**Results:**

All patients showed excellent results in SST (99.4% ± 0.02), SSV (98.3% ± 0.04), ASES-score (100% ± 0) and PASS (0.99 ± 0.01). In the DASH-score, 17 patients had excellent results, one patient showed a good outcome. Fracture healing occurred in all patients without complications. Eight patients complained about bothering scars. Age, gender and fracture morphology did not affect the outcome. Revision surgery after secondary fracture dislocation did not show a worsened outcome compared to primary plate fixation. Physeal growth plate bridging implants did not worsen the outcome. The timing of implant removal within the first 6 months postoperatively did not affect long-term function.

**Conclusion:**

Plate fixation is a safe option in pediatric patients with limited growth potential and highly displaced PHFs. Plate fixation led to a good to excellent functional outcome, regardless of fracture morphology and implant positioning. A higher invasiveness and the need for implant removal must be considered.

## Introduction

**Fig. 1:**
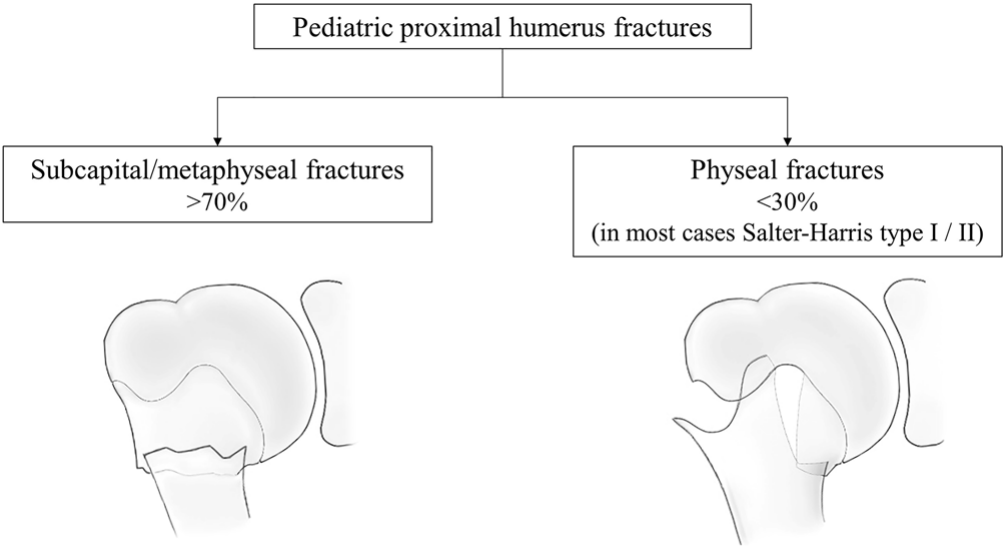
Fracture types of pediatric proximal humerus fractures [4, 5]

In pediatric trauma, fractures of the proximal humerus are a comparably rare entity, accounting for about 2% of all pediatric fractures[1, 2]. A peak incidence can be found in early adolescence between 10 and 14 years of age^3^, ^4^. Fracture types range from metaphyseal in the majority of cases to physeal fractures, especially Salter–Harris I and II fractures (see Fig. 1) [4, 5].

Since the proximal humeral growth plate is responsible for about 80% of humeral growth, it provides a high potential for remodelling [6–8]. Physiologically, a humeral growth spurt can be observed in females between 11 and 13, and males between 13 and 15 years of age [9]. Closure of the proximal humeral growth plate gradually occurs between 14 and 17 years of age. Females usually reach skeletal maturity one to two years prior to males [6, 7, 10, 11].

Due to the individual, age-dependent remodelling capacities and the heterogenous fracture patterns, the adequate treatment of pediatric proximal humerus fractures (PHF) still is a matter of discussion and therapeutic recommendations widely differ. In non-displaced fractures, non-operative treatment is recommended. Especially in patients ≤ 10 years of age, moderate dislocation can be compensated without causing functional impairments [2, 4, 12–14]. In a study including pediatric PHFs regardless of age and fracture morphology, Lähdeoja et al. even stated that an operative therapy generally does not improve the patients’ long-term outcome [15].

In more differentiated approaches, recent studies underline the importance of operative treatment, especially in adolescent patients with displaced or non-reducible fractures [12, 16–19]. Chaus et al. showed that with every year of age, the odds ratio for a less than desirable outcome increases by the factor 3.81 [18]. For highly displaced fractures, Pavone et al. described a better outcome after open reduction techniques compared to closed reduction and internal fixation [17]. Shariah et al. recommended surgical treatment in angulation > 40° in patients with less than two years of remaining growth. Other studies including Binder et al. favour operative anatomical reduction in ≥ 12 year-old patients and fracture angulation > 20° due to the high risk of soft tissue interposition [9, 12, 16].

Closed procedures with percutaneous K-wire pinning or elastic stable intramedullary nailing (ESIN) provide similarly excellent results [16–23]. However, several studies describe interposition of the long head of the biceps (LHB) tendon, periosteum, the deltoid muscle, the conjoint tendons or comminuted, bony fragments, preventing closed reduction [12, 20, 24]. In these cases, conversion to open reduction is indicated. Since in some severely displaced fractures, loss of reduction after retention by K-wires or ESINs are described, plate- and screw-fixation can also be performed in open approaches [17].

Studies analysing the outcome following plate fixation of pediatric PHFs are lacking [23]. In a case series including 6 patients, Freislederer et al. described excellent outcomes in a two-year follow-up period after plate fixation [24]. In several other studies, specific cases of plate fixation with very good functional results are described, the low numbers of patients, however, do not allow statistical conclusions [17, 19, 25].

We hypothesised, that open reduction and plate fixation in pediatric PHFs is a reliable treatment option in case of insufficient closed reduction and severely displaced fracture patterns with a high risk of soft tissue interposition.

The study was approved by the institutional review board (IRB No. 2019-627-f-S, University of Münster, Germany).

## Materials and methods

**Fig. 2:**
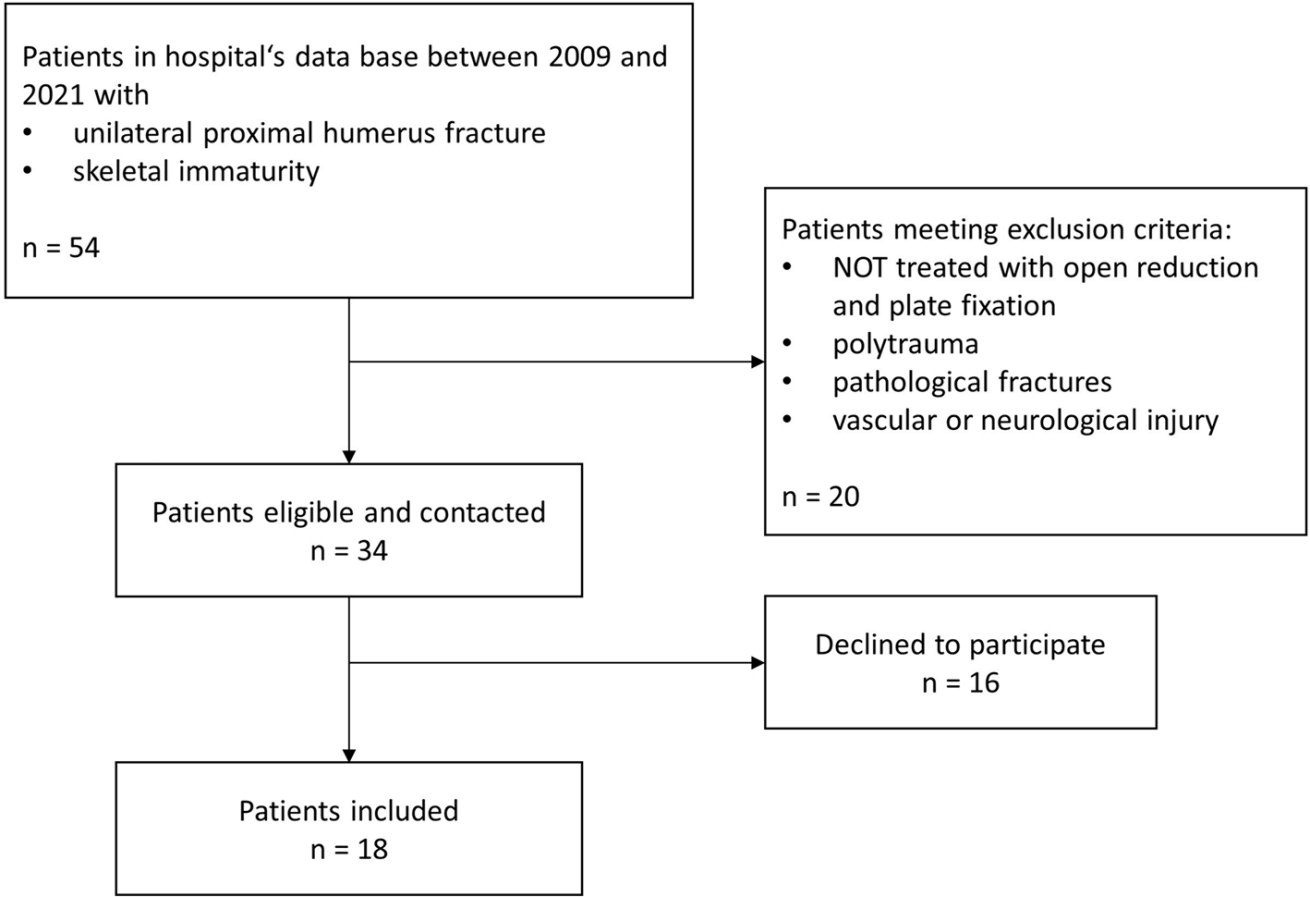
Participant flow diagram showing numbers of patients eligible, contacted and finally included in this study

In this retrospective, single-center series, pediatric PHFs treated with open reduction and internal plate fixation within the authors’ level 1 trauma surgery department between 2009 and 2021 were analysed. Skeletally immature patients with unilateral, dislocated PHFs treated with plate fixation were included. Exclusion criteria were the presence of pathological fractures, polytrauma patients and concomitant vascular or neural injury. After applying these criteria, 34 patients were contacted, of whom 18 patients agreed on participating in this study (see Fig. 2).

Within these 18 patients, there were ten female and eight male patients. The mean follow-up was 78.2 months (8–157 months). The mean patients’ age at trauma was 12.1 years (± 2.4; 5–16 years) with one patient below 10 years of age. In 17 patients, there were no relevant pre-existing conditions, while one patient suffered from a statomotor retardation and implantation of a cardiac pacemaker had been performed previously. Among all patients, a total of 12 experienced senior surgeons performed surgery.

Regarding fracture morphology, the Salter–Harris (SH) classification was applied to all physeal fractures [26]. All fractures were additionally classified by the Neer–Horwitz classification: I: no displacement; II: displacement less than one-third of the shaft width; III: displacement less than two-thirds of the shaft width; IV: displacement greater than two-thirds of the shaft width [27].

### Surgical technique

**Fig. 3:**
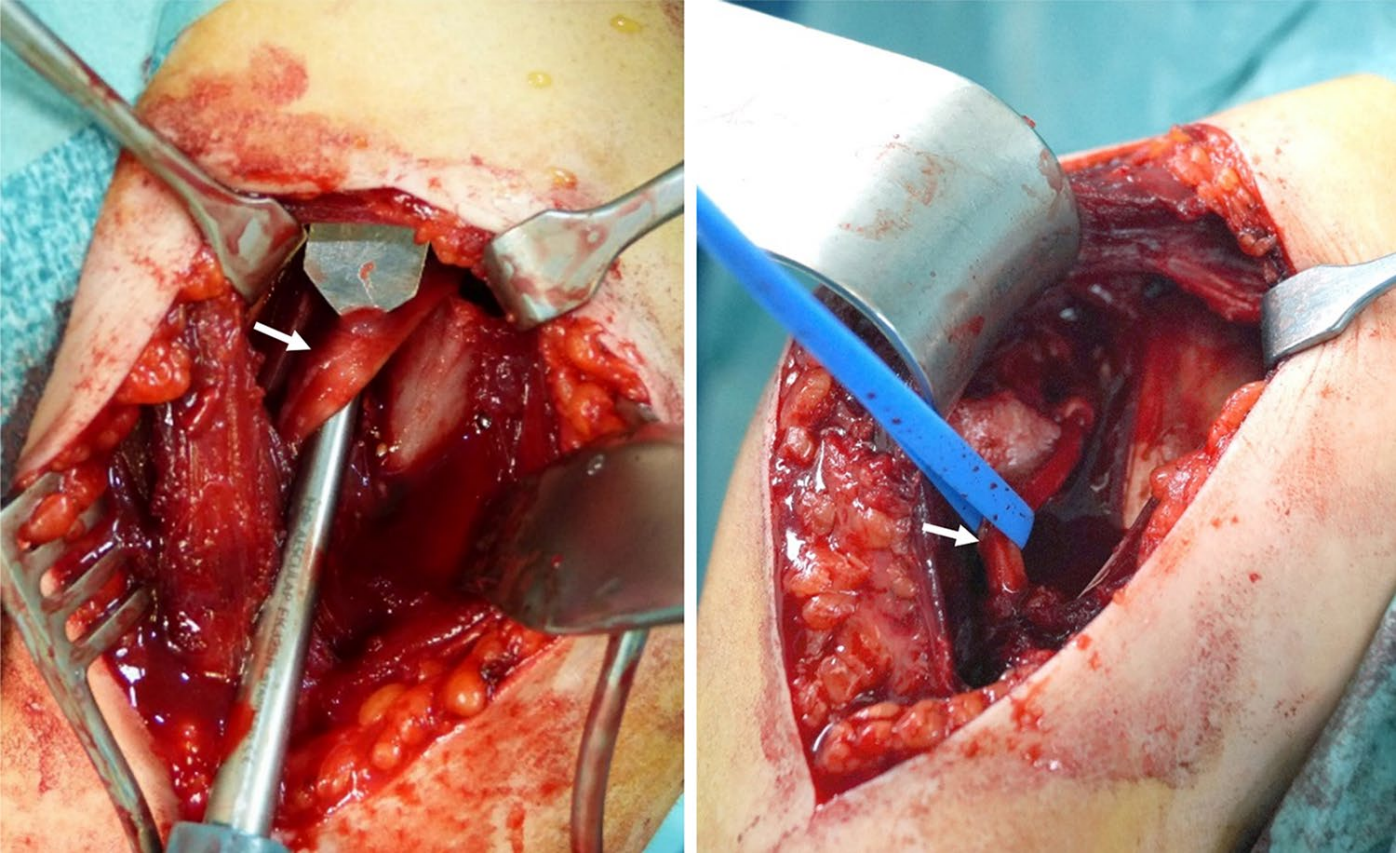
Intraoperative findings of a 15-year-old male patient (left) and an 11-year-old female patient (right) with interposition of the long head biceps tendon (white arrows) within the proximal humeral fracture, preventing closed reduction

**Fig. 4:**
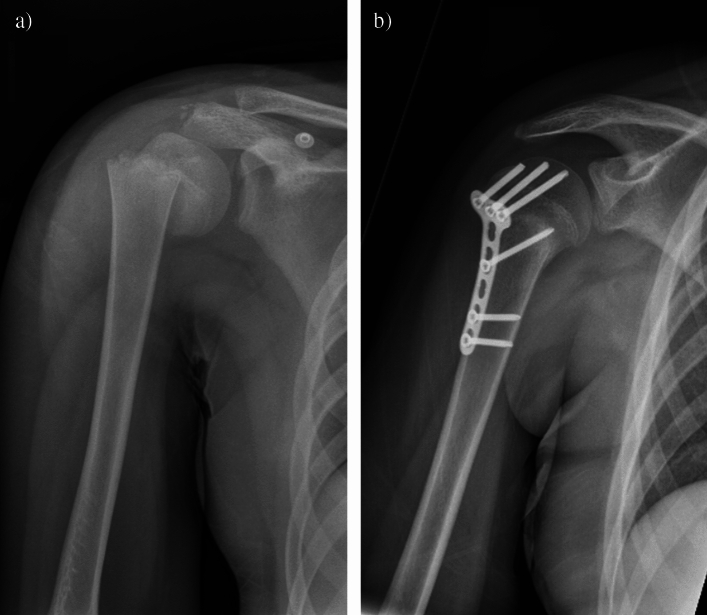
(**a**) Radiograph of a 14-year-old male patient following closed K-wire fixation in an external hospital and secondary dislocation. (**b**) Postoperative radiograph four weeks after revision surgery including K-wire removal, open reduction and plate fixation

**Fig. 5:**
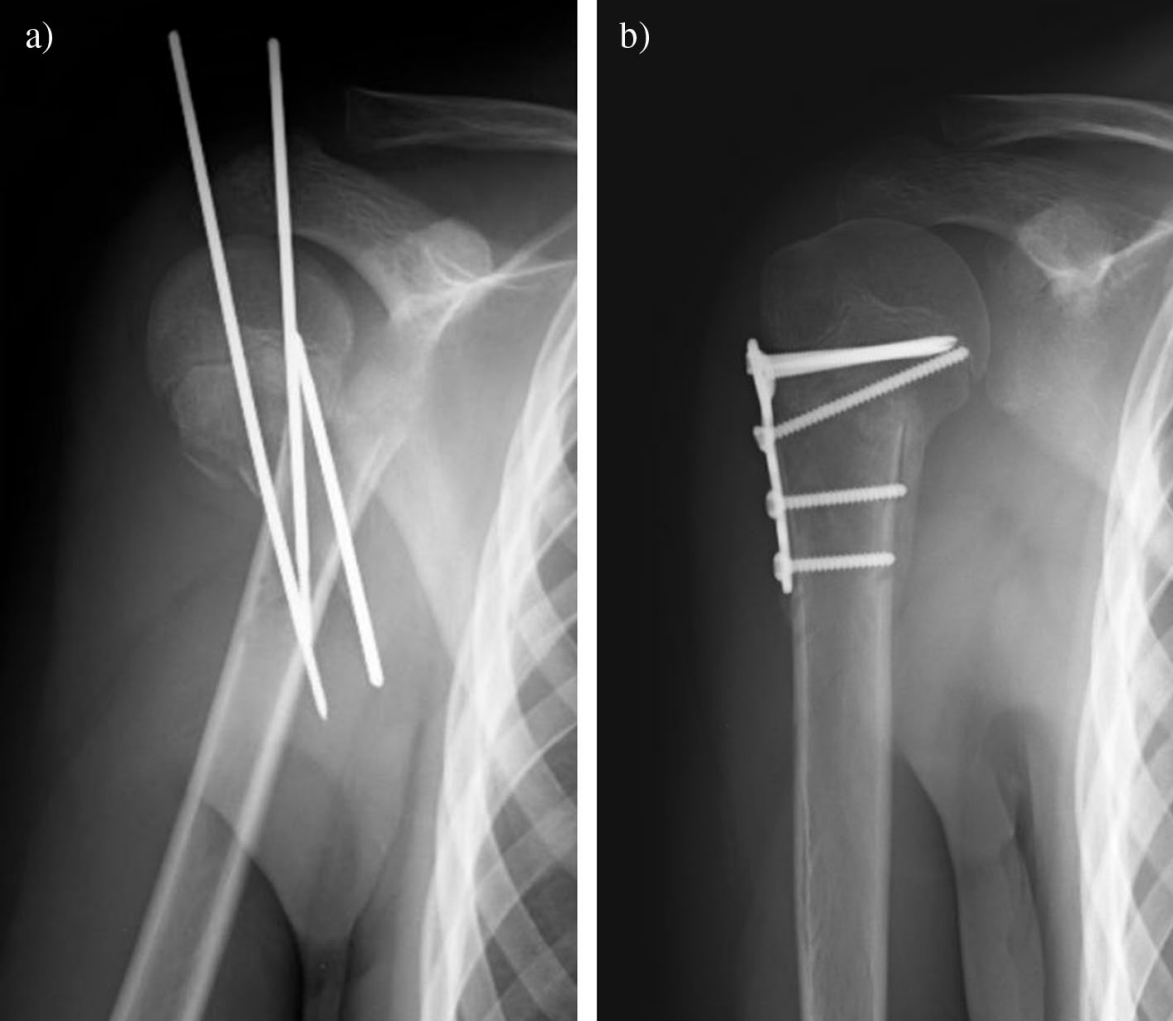
(**a**) Radiograph of a 12-year-old female patient presenting with a proximal humerus fracture (Salter–Harris type II) in varus displacement. (**b**) Postoperative radiograph following open reduction and angular stable plate fixation via a deltopectoral approach

Surgery was performed in a beach chair position under general anaesthesia. In all primary fractures closed reduction failed and an open procedure was performed. In two cases of revision due to secondary dislocation after K-wire fixation (n = 1) and ESIN implantation (n = 1), open surgery was performed directly. In 17 patients the deltopectoral approach was used, while in one patient a delta-split approach was performed. After visualizing the fracture, entrapped soft tissue within the fracture was released if necessary (see Fig. 3). The fracture was reduced under image intensifier and temporarily fixated via K-wires. Final retention was achieved by plate fixation. Different plates were used (3.5 mm T-plate in n = 12; 2.7 mm T-plate in n = 4; 2.4 mm LCP in n = 1; 3.5 mm PHILOS-plate in n = 1) and individually contoured (see Figs. 4 and 5). In 12 patients, angular stable fixation was performed. One patient required implantation of an additional screw. Plates were either positioned growth plate spanning with screws in both, epiphyseal and metaphyseal bone (in n = 11 patients), or distal to the growth plate (in n = 7 patients). Growth plates were not directly penetrated by implants in any patient. Finally, the periosteum was sewed. Postoperative care included early functional treatment without the use of immobilizers and casts. No weight bearing was recommended for four to six weeks postoperatively.

### Clinical assessment

Since an assessment of glenohumeral motion is not reliable perioperatively in children with acute injury, the clinical assessment was performed postoperatively by one experienced examiner (I.V.).

Regarding the active range of motion (ROM), the injured shoulder was compared to the healthy shoulder. Measurements were performed with a goniometer. Radiographs were analysed regarding implant positioning, dislocation and fracture consolidation.

For subjective assessment a visual analogue scale (VAS) was used for pain at rest and under strain. Patients were questioned about subjective complaints as functional and cosmetic impairments.

Different clinical scores were assessed, including the Disabilities of the Arm, Shoulder and Hand (DASH) Score [28, 29], the Simple Shoulder Test (SST) [30], the Subjective Shoulder Value (SSV) [31], the American Shoulder and Elbow Surgeons (ASES) shoulder score [29, 32] and the Pediatric and Adolescent Shoulder Survey (PASS) [33]. The scores were interpreted by the Cicchetti’s criteria: less than 0.40 is poor, 0.40–0.59 is fair, 0.60–0.74 is good, and 0.75–1.00 is excellent [34].

### Statistical analysis

Subgroup analyses were either performed by unpaired t-tests or one-way ANOVA with Tukey correction for multiple comparisons, using the GraphPad Prism 9 software (GraphPad Software, San Diego, CA, USA). All analyses were performed with a significance level of p < 0.05 and a confidence level of 95%. For correlation analyses, a linear regression model was applied, and the determination coefficient (R^2^) as well as the p value was used to measure correlation.

## Results

Nine patients presented with physeal fractures (n = 2 with SH type I, n = 7 with SH type II), while n = 9 PHFs were subcapital or metaphyseal fractures. Among all n = 18 fractures, there were n = 6 NH type II, n = 7 NH type III and n = 5 NH type IV fractures.

Mean fracture angulation was 32.3° (± 10.89°; 20–53°). In 15 patients, surgical treatment was performed primarily. One patient was treated non-operatively first and showed secondary fracture dislocation one week after trauma. Two patients presented with secondary fracture dislocation after surgical treatment (n = 1 following ESINs, n = 1 following K-wire fixation).

The mean time of surgery was 92.17 min (± 43.89). Intraoperatively, soft tissue interposition within the fracture was seen in nine patients. The LHB tendon was entrapped in six patients, periosteal interposition was seen in five patients, while in one patient entrapment of the deltoid muscle was observed. In three of these patients a combination of LHB tendon, periosteum or deltoid muscle interposition occurred.

During the follow-up period, no surgical revision after plate fixation was necessary. Postoperative infection did not occur and in radiological assessment, no fracture dislocation and no implant failure were observed. Regular bone healing leading to complete fracture consolidation was seen in all 18 patients.

Removal of implants was performed in 17 of 18 patients after a mean time of 4.76 months (± 3.59) following plate fixation. One patient was referred to an external hospital for implant removal. The earliest removal of implants was performed 60 days postoperatively, while there was a maximum of 16.37 months until the implants were removed.

In the assessment of ROM after a mean clinical follow-up time of 6.52 years (± 4.37, 0.67–13.08), mild impairments were observed in six patients compared to the respective, contralateral, healthy shoulder. Four patients suffered from deficits in external rotation (n = 3 with a deficit of 10°, n = 1 with a deficit of 20°). However, the comparison between n = 18 fractured, and n = 18 healthy shoulders regarding external rotation showed no significant difference between both groups (p = 0.056). In two patients, shoulder extension was reduced by 10° and 20°, respectively. A 15° reduction of adduction was observed in one patient. Glenohumeral flexion, abduction and internal rotation was not impaired in any patient. The postoperative follow-up time did not differ significantly between patients with and without ROM impairments (p = 0.8624). Neurologically, one patient described localized hypaesthesia adjacent to the scar. Further neurological complications were not observed.

Subjectively, eight patients described cosmetically bothering scars, one patient suffered from weather sensitivity. Four patients described mild, subjective, functional impairment, for example a mild reduction of strength or mild deficits in extensive rotational movements. A regular return to sports and overhead activities was seen in all patients. All patients described an excellent, subjective satisfaction with the postoperative outcome.

The results of all applied clinical scores among all n = 18 patients are summed up in Table 1.

**Table 1:** Evaluation of clinical scores in the entire study population with n = 18 patients

Score	Mean (n = 18)	Range (Min–Max)	Evaluation
Pain at rest (VAS)	0	0	Excellent (n = 18)
Pain under strain (VAS)	0	0	Excellent (n = 18)
SST	100%	90–100%	Excellent (n = 18)
SSV	98.28%	85–100%	Excellent (n = 18)
ASES score	100	100	Excellent (n = 18)
PASS	0.9933	0.97–1	Excellent (n = 18)
DASH score	1.4/100	0–25/100	Excellent (n = 17) Good (n = 1)

Mean values of the Visual Analogue Scale (VAS) for pain at rest and under strain, as well as the Simple Shoulder Test (SST), Subjective Shoulder Value, the American Shoulder and Elbow Surgeons (ASES) shoulder score, the pediatric and adolescent shoulder survey (PASS) and the disabilities of arm, shoulder and hand (DASH) score are described

### Subgroup analyses

For subgroup analyses, we included the SSV and PASS, showing the most differentiated distribution of score values. Results are described in Table 2.

**Table 2:** Subgroup analyses regarding the Pediatric/Adolescent Shoulder Survey (PASS) and the Subjective Shoulder Value (SSV)

	PASS		SSV	
	Mean score (± SEM)	p value	Mean score (± SEM)	p value
Fracture localization				
Salter–Harris I/II (n = 9)	0.994 ± 0.002	*0.59*	98.78 ± 1.1	*0.63*
Subcapital/metaphyseal (n = 9)	0.992 ± 0.004		97.78 ± 1.69	
Fracture morphology				
Neer–Horwitz II (n = 6)	0.993 ± 0.005	≥ *0.9*	97.5 ± 2.5	≥ *0.88*
Neer–Horwitz III (n = 7)	0.994 ± 0.002		98.57 ± 1.43	
Neer–Horwitz IV (n = 5)	0.992 ± 0.004		98.8 ± 0.97	
Age				
< 12-year-old (n = 5)	0.998 ± 0.002	*0.15*	98 ± 2	*0.87*
≥ 12-year-old (n = 13)	0.992 ± 0.002		98.38 ± 1.18	
Gender				
Female (n = 10)	0.991 ± 0.004	*0.58*	97.40 ± 1.7	*0.33*
Male (n = 8)	0.994 ± 0.003		99.38 ± 0.63	
Primary ORIF (n = 15)	0.993 ± 0.002	> *0.99*	98.93 ± 0.998	*0.14*
Revision surgery (n = 3)	0.993 ± 0.007		95.00 ± 2.9	
Positioning of implants				
Growth plate bridging (n = 11)	0.996 ± 0.002	*0.05*	99.91 ± 0.09	0.03 (*)
Distal of growth plate (n = 7)	0.989 ± 0.004		95.71 ± 2.3	
Time to implant removal				
≤ 3 months (n = 7)	0.992 ± 0.003	≥ *0.67*	99.14 ± 0.7	≥ *0.71*
3–6 months (n = 7)	0.991 ± 0.004		97.86 ± 2.14	
> 6 months (n = 3)	0.997 ± 0.003		96.67 ± 3.33	

Mean score values and standard errors of mean (SEM) are described. Unpaired t-tests or one-way ANOVA with Tukey correction for multiple comparisons were applied with a statistical significance level of p < 0.05 (*)

Fracture morphology, age, gender and time of implant removal did not impact the functional outcome significantly. Outcomes after revision surgery did not differ compared to primary plate fixation. Regarding plate positioning, the PASS score did not differ between physeal growth plate bridging plate fixation and plate positioning distal to the physeal growth plate. Regarding the SSV, a significantly higher mean value was found in patients with physeal growth plate bridging implants. However, the SSV’s minimal clinically important difference (MCID) was not matched [35].

Considering fracture angulation, the correlation between initial angulation and external rotation, SSV and PASS score was very low with determination coefficients of R^2^ ≤ 0.14 and p values ≥ 0.1277.

## Discussion

In this case series of pediatric PHFs, we can summarize the following main findings: plate fixation is a safe option in pediatric patients with highly dislocated PHFs or in cases of surgical revision. Regardless of the patients’ age and gender, fracture morphology and implant positioning, plate fixation led to a good to excellent functional outcome in all patients included in this study.

In literature, standardized therapeutic algorithms for pediatric PHFs have not been established, yet. Dobbs et al. suggested a liberal, non-operative approach, accepting fracture angulation up to 60° in patients with 8–11 years of age and angulation up to 45° in ≥ 12-year-old patients [14]. In more recent studies and meta-analyses recommendations shifted towards surgical treatment, narrowing indications for non-operative approaches. Lefèvre et al. performed fracture reduction in 10 to 13-year-old patients with translation of > 50% or angulation > 40° and in > 13-year-old patients with translation > 30% an angulation > 20°[4]. Shahriar et al. suggested operative treatment in patients with less than 2 years of remaining skeletal growth and fracture angulation > 40°, considering the gender-specific skeletal maturation [9, 10]. Binder et al. recommended surgical, anatomical reduction in > 12 year-old patients and > 20° angulation due to limited remodelling potential and the high risk of soft tissue interposition [12].

In the analysed study population, a rather aggressive surgical approach was used, including open, anatomical reduction and plate fixation. All patients presented with a fracture angulation ≥ 20°, while 17 of 18 patients were ≥ 10 years of age. One 5-year-old patient presented with a fracture angulation of 39° and severe rotational malalignment. The patient was referred to our department from an external hospital and, therefore, surgery was performed not until the third day after trauma. These circumstances led to the decision of performing open fracture reduction combined with plate fixation due to the rigidly dislocated fracture. Still, open plate fixation in children below ten years of age remains debateable and is usually not required.

Generally, plate fixation is a more invasive approach, compared to ESIN or K-wire fixation. Regarding the time of surgery and fluoroscopy time, a comparison of these methods is difficult due to limited data published. Pavone et al. and Wang et al. presented cohorts, in which K-wire fixation was performed within a mean time of 28 and 69.07 min, respectively [17, 36]. In their studies, however, the majority of fractures was suitable for closed reduction and percutaneous fixation. For severely displaced fractures, Hutchinson et al. separately analysed open reduction techniques and described mean OR times of 90 and 174 min for open K-wire fixation and open intramedullary nailing, respectively [37]. Compared to these differentiated data, plate fixation does not relevantly increase the operating time if the fracture morphology requires open reduction.

Regarding the fluoroscopy time, comparable data are lacking. Due to the direct visualization of fracture reduction, we assume that the intraoperative exposure to radiation is reduced, compared to closed and mini-open procedures. Future studies with larger patient cohorts and multicenter study designs should help to clarify this assumption.

Postoperatively, plate fixation allows safe, early functional rehabilitation, however, implant removal is required, making a second operative procedure necessary. Plate removal leads to more soft tissue trauma and usually requires general anaesthesia and a longer operation time compared to ESIN or K-wire removal. In contrast, percutaneous K-wire fixation can lead to soft tissue or neural irritation causing pain and impaired ROM [16, 21, 38].

In this study, the main subjective disadvantages were cosmetically bothering scars, which should not be undervalued in a pediatric patient cohort. Regarding the functional outcome, all patients presented with reliable, reproducible as well as excellent results, independent of age, gender and initial fracture angulation. During skeletal growth, implant removal should be performed as soon as fracture healing has occurred. As this study suggests, plate removal can usually be performed three months after plate fixation. Also, results were excellent after plate removal within a period of three to six months postoperatively, so that implant removal could also be postponed, if there is any doubt regarding fracture stability.

Regarding plate positioning, growth plate spanning implants did not lead to functional impairments. A higher mean SSV was seen compared to patients with implants distal to the growth plate, the SSV’s MCID was not matched, though [35]. We consider this difference to be mainly caused by the small study group. However, it may underline the importance of anatomical reduction and allows the use of growth plate spanning implants, if required by the fracture’s morphology.

Since no difference between revision surgery and primary plate fixation was seen, we consider open plate fixation to be a safe option in case other fixation methods fail. Still, future studies require larger patient collectives including revision cases to confirm this assumption.

The validity of the applied clinical scores can be considered a limitation, as the SST, SSV and ASES score are not specifically validated for pediatric patients [29–31]. While the DASH score is validated for numerous upper extremity injuries, it does not represent specific shoulder joint function [28, 29, 39, 40]. Since the PASS is one of the rare scoring systems validated for pediatric patients, we mainly used PASS values for subgroup analyses [33]. Also, we considered the SSV to be useful in a pediatric patient collective, due to its simple assessment.

The low number of patients and the retrospective study design without control group must be mentioned as limitations, as well. Also, the high number of twelve different surgeons involved in surgical treatments must be considered. Since the surgical techniques were similar and all patients were treated in the same level 1 trauma department by senior surgeons, a reliable level of comparability can be assumed.

The incidence of long-term growth disturbances has not been evaluated in all patients due to a follow-up range of 8–157 months. Since PHFs in pediatric patients are rare and treatment options differ, large studies regarding open plate fixation of pediatric PHFs are lacking. Considering previous studies which include plate fixation methods [17, 19, 24, 25], this study provides a comparably high number of patients treated with open plate fixation.

Finally, further studies including control groups and a higher number of patients are required. Especially in cases of complex fracture morphology or revision surgery, valid comparisons between open and closed reduction, as well as between ESIN, K-wire and plate fixation are desirable.

## Conclusion

In pediatric PHFs, open reduction and plate fixation is a safe and stable fixation method leading to a very good postoperative outcome, regardless of age, gender, fracture morphology and angulation. Cases of revision surgery presented with equally high functional outcome scores, compared to primary plate fixation. However, a higher invasiveness and the need for implant removal must be considered. We suggest that plate fixation can be considered as a therapeutic option in complex, pediatric PHFs or if less invasive methods of fracture reposition and retention fail.

## Data Availability

The data that support the findings of this study are not openly available due to reasons of sensitivity and are available from the corresponding author upon reasonable request.
